# Editorial: Advancing our understanding of the genetic and functional basis of skeletal dysplasia

**DOI:** 10.3389/fgene.2023.1139228

**Published:** 2023-01-27

**Authors:** Long Guo, Rong Qiang, Yi Zhang, Pelin Ozlem Simsek-Kiper

**Affiliations:** ^1^ Department of Laboratory Animal Science, School of Basic Medical Sciences, Xi’an Jiaotong University, Xi’an, China; ^2^ Shaanxi Institute for Pediatric Diseases, Xi’an Children’s Hospital, Affiliated Children’s Hospital of Xi’an Jiaotong University, Xi’an, China; ^3^ Center of Medical Genetics, Northwest Women’s and Children’s Hospital, Xi’an, China; ^4^ Department of Orthopaedics, Xiangya Hospital, Central South University, Changsha, China; ^5^ Hacettepe University Faculty of Medicine, Department of Pediatrics, Pediatric Genetics Unit, Ankara, Türkiye

**Keywords:** skeletal dysplasia, genetics, development, WES, WGS

Skeletal dysplasia is a group of disorders characterized by abnormal skeleton formation because of intrinsic derangement of growth, development, and/or differentiation. According to the latest International Classification of Skeletal Dysplasia, 42 subgroups with 461 disease entities have been established in this category. More than 90% of skeletal dysplasias are monogenic diseases. The incidence of each disease is low, but as a whole, skeletal dysplasias affect about one in 1,000 of the global population. Therefore, studies on skeletal dysplasia have essential clinical significance (*e.g.,* prenatal diagnosis, targeted therapy, prevention at the second pregnancy) and provide the highest evidence for the mechanism of development and maintenance of the human skeleton.

For this Research Topic, we collected high-quality research papers describing novel insights into genetic factors that decide heterogeneity, prognosis, and treatment of skeletal dysplasias. The final Research Topic has 14 published articles covering various types of skeletal dysplasias in different populations.

Osteogenesis imperfecta (OI) is a rare inherited connective tissue dysplasia characterized by skeletal fragility, recurrent fractures, and bone deformity, predominantly caused by mutations in *COL1A1* or *COL1A2* that encode the chains of type I collagen. Although mutation spectrums on autosomal dominant OI have been established in large cohorts of Chinese populations, the relationship between clinical manifestations and genetic mutations remains to be further explored due to the high genetic and clinical heterogeneity. Chen et al. performed a detailed analysis on 187 Chinese OI patients to further expand the mutational spectrum of type I collagen genes, and better establish the correlation between genotype and phenotype in OI patients. The findings coupled with the heterogeneity observed in the transcriptomic data derived from osteoblasts of six patients from the cohort. Notably, they highlighted that bisphosphonate treatment benefits bone density rather than height during the juvenile stage (10–15 years old). Observing effective bisphosphonate treatment in an age-specific manner would help improve OI patient management.

Dysosteosclerosis (DOS) is a rare sclerosing bone dysplasia characterized by unique osteosclerosis of the long tubular bones and platyspondyly. DOS is inherited in an autosomal recessive manner and is genetically and clinically heterogeneous. Pathogenic variants in *SLC29A3, TNFRSF11A, TCIRG1, LRRK1,* and *CSF1R* have been reported to be associated with DOS. Mutations in *TNFRSF11A* encoding RANK protein also cause osteopetrosis, autosomal recessive 7 (OPTB7). Based on the previous studies, it is hypothesized that mutations producing aberrant mutant RANK proteins (missense or truncated or elongated) cause DOS, while null mutations lead to OPTB7. Kırkgöz et al. presented the fifth case of TNFRSF11A-associated DOS with a novel homozygous frame-shift mutation. The mutation is predicted to cause non-sense mutation-mediated mRNA decay in all RANK isoform transcripts, resulting in a totally null allele. The finding opposes the previous hypothesis and suggests that the genotype-phenotype relationship in TNFRSF11A-associated OPTB7 and DOS remains unclear. Further studies are necessary to understand the phenotypic spectrum caused by *TNFRSF11A* mutations.

Congenital contractural arachnodactyly (CCA) is a rare autosomal dominant disorder of connective tissue characterized by crumpled ears, arachnodactyly, camptodactyly, large joint contracture, and kyphoscoliosis. CCA is caused by variants in *FBN2* encoding fibrillin-2, which is an integral component of elastin fibers in the extracellular matrix. The natural course of CCA has yet to be well-described. To decipher the genetic and phenotypic spectrum of CCA. Sun et al. enrolled a CCA cohort *via* the DISCO consortium (http://www.discostudy.org/) and identified ten pathogenic *FBN2* variants in 27 CCA patients from ten families. Among these ten variants, seven were novel. They also validated the clinical utility of a newly developed scoring system for CCA.

Polydactyly is a common congenital abnormality characterized by the presence of extra digit(s) on the preaxial or postaxial sides of the hand or foot. A complex network of intercellular communication and gene expression regulates limb growth in vertebrates. The sonic hedgehog protein (SHH), one of three mammalian hedgehog proteins, is expressed in the zone of polarizing activity (ZPA) of the limb bud and those of the notochord and floor plate in the neural tube, playing an essential role in regulating the patterning and growth of the developing limb. ZPA regulatory sequence (ZRS) is a limb-specific enhancer of *SHH*, located nearly 1 Mb from *SHH* and within intron 5 of *LMBR1*. Zeng et al. recruited 167 sporadic or familial cases (including 154 sporadic patients and 13 families) with preaxial polydactyly from Central-South China and identified four ZRS variants in four patients (2.40%, 4/167). This investigation preliminarily evaluated a ZRS variants rate in patients with preaxial polydactyly and described the general picture of preaxial polydactyly in Central-South China. Smoothened (SMO) is one of the significant components of the SHH pathway. Fan et al. identified bi-allelic novel variants of SMO in a patient with postaxial polydactyly and documented the detailed phenotype. These findings highlight the importance of the SHH pathway in human limb patterning by studying the etiology of polydactyly and contribute to a better understanding of the complex phenotypes and mechanisms associated with the defects of the SHH pathway.

Spondylo-epi-metaphyseal dysplasia (SEMD) is a heterogeneous group of disorders with different modes of inheritance and is characterized by disproportionate or proportionate short stature. More than 30 disease-causing genes have been identified to date, and different types of SEMD exhibit considerably overlapping clinical features, which usually complicate the diagnosis. Lv et al. enrolled seven families, including 11 patients with SEMD, and analyzed their clinical, radiographic, and genetic features. Seven variants were identified in *TRPV4, COL2A1, CCN6, SBDS*, and *ACAN*. In addition, Fan et al. reported a novel splicing variant of *COL2A1* in a Chinese family with SEMD and confirmed the pathogenicity of the variant by a minigene analysis. These studies expand the phenotypic and genetic spectrum of SEMD and provide evidence for the genotype-phenotype relations, contributing to molecular and clinical diagnosis of SEMD.

Kashin–Beck disease (KBD) is an endemic osteoarthropathy distributed throughout North Korea, Siberia, Japan, and China. Selenium deficiency and T-2 toxin have been considered the main environmental risk factors for KBD that induce cartilage damage, such as acceleration of chondrocyte apoptosis and an imbalance of the extracellular matrix. However, the role of selenium deficiency and T-2 toxin in KBD development remains unclear. Ning et al. took the cubital venous blood of 258 subjects including 129 sex-matched KBD patients and 129 healthy controls for single nucleotide polymorphisms (SNPs) detection and analysis. They selected these candidate SNP loci from selenium- and T-2 toxin–responsive genes in Comparative Toxicogenomics Database and verified the gene expression in knee cartilage of the patients and controls. These findings revealed the interaction between genetic and environmental factors and suggest that genomic variation in selenium deficiency- and T-2 toxin-responsive genes increase the risk of KBD by disturbing extracellular matrix homeostasis.

The Research Topic also included reports about acromesomelic dysplasia, Maroteaux type (Wu et al.), X-linked hypophosphataemia (Yang et al.), Aarskog–Scott syndrome (Zhu et al.), campomelic dysplasia (Calvache et al.), and spondylocostal dysostosis (Umair et al.) They either uncovered new genotype-phenotype associations by presenting exceptional cases or summarized the study progression of these skeletal dysplasias by designing a comprehensive review. Additionally, Toor et al. revealed novel differentially expressed coding genes and LncRNAs by profiling the transcriptome of murine osteoclast differentiation.

The above reports enrich our knowledge of skeletal dysplasia by reporting either large cohorts or atypical cases. These findings greatly expand the phenotypic and mutational spectrum, and contribute to the diagnosis, prevention and therapeutic innovation of skeletal dysplasia. These studies also provide insight into the underlying pathologic mechanisms, gene-environment interactions, and skeletal developmental biology. We sincerely appreciate the time and effort of all the authors, who contributed to the Research Topic, which significantly improves our understanding of the genetic and functional basis of the skeletal dysplasias.

